# Underused Marine Resources: Sudden Properties of Cod Skin Gelatin Gel

**DOI:** 10.3390/gels9120990

**Published:** 2023-12-18

**Authors:** Yuriy F. Zuev, Svetlana R. Derkach, Liliya R. Bogdanova, Nikolai G. Voron’ko, Yulia A. Kuchina, Aidar T. Gubaidullin, Ivan V. Lunev, Oleg I. Gnezdilov, Igor A. Sedov, Radik A. Larionov, Larisa Latypova, Olga S. Zueva

**Affiliations:** 1 Kazan Institute of Biochemistry and Biophysics, FRC Kazan Scientific Center of RAS, 2/31 Lobachevsky Street, 420111 Kazan, Russiaa_t_gubaidullin@mail.ru (A.T.G.); lounev75@mail.ru (I.V.L.); igor_sedov@inbox.ru (I.A.S.); 2 Laboratory of Chemistry and Technology of Marine Bioresources, Institute of Natural Science and Technology, Murmansk State Technical University, 183010 Murmansk, Russia; derkachsr@mstu.edu.ru (S.R.D.); voronkonikolay@mail.ru (N.G.V.); kuchinayua@mstu.edu (Y.A.K.); 3 Arbuzov Institute of Organic and Physical Chemistry, FRC Kazan Scientific Center of RAS, 8 Arbuzov Street, 420088 Kazan, Russia; 4 Institute of Physics, Kazan Federal University, Kremlyovskaya St.18, 420008 Kazan, Russia; goi@yandex.ru (O.I.G.); radik.larionov@gmail.com (R.A.L.); 5 School of Chemistry and Chemical Engineering, Harbin Institute of Technology, 92 West Da-Zhi Street, Harbin 150001, China; larisa.latypova@hit.edu.cn; 6 Institute of Electric Power Engineering and Electronics, Kazan State Power Engineering University, 51 Krasnoselskaya Street, 420066 Kazan, Russia; ostefzueva@mail.ru

**Keywords:** gelatins, fish, mammalians, gels, structure, physicochemical properties

## Abstract

The main object of this work was to characterize the structure and properties of laboratory-made fish gelatin from cod skin in comparison with known commercial gelatins of fish and mammalian origin. This is one way we can contribute to the World Food Program and characterize foodstuff resources from alternative natural sources. Our research was based on the combination of an expanded set of complementary physical–chemical methods to study the similarities and distinctions of hydrogels from traditional and novel gelatin sources from underused marine resources. In this work, we have compared the morphology, supramolecular structure and colloid properties of two commercial (mammalian and fish) gelatins with gelatin we extracted from cold-water cod skin in laboratory conditions. The obtained results are novel, showing that our laboratory-produced fish gelatin is much closer to the mammalian one in terms of such parameters as thermal stability and strength of structural network under temperature alterations. Especially interesting are our experimental observations comparing both fish gelatins: it was shown that the laboratory-extracted cod gelatin is essentially more thermally stable compared to its commercial analogue, being even closer in its rheological properties to the mammalian one.

## 1. Introduction

Gelatin is the protein compound obtained as a result of digestion of native collagen contained in the bones, cartilage, and skin of mammals and fish [1,2,3]. The type of raw material used and its chemical composition are important factors affecting the properties of gelatin. The most common sources of gelatin for industrial production are pork and bovine skins and the bones of pigs and cattle [4,5,6]. In recent years, there has been a significant increase in the production of gelatin from alternative sources, such as raw fish materials [7,8,9,10,11]. In addition to the well-known sociocultural and veterinary–sanitary aspects such as the current state of affairs, requirements for the rational utilization of waste from the fishing industry are the reasons for increased interest in fish gelatin study and applications [12,13,14,15].

The collagen molecule (tropocollagen) is a left-handed helix composed of three α-chains linked by covalent bonds [16]. The transformation of collagen to gelatin is a process in which a highly organized, water-insoluble collagen fiber transforms from an endless asymmetric network of interconnected tropocollagen units into simpler structural units with a lower degree of internal order. The degree of collagen conversion to gelatin depends on the pretreatment stage and on the conditions of the extraction process (pH, temperature, and extraction time) [17,18,19]. Depending on the stage of pretreatment, two types of gelatins are distinguished: type A gelatin (isoelectric point at pH 7–9) and type B gelatin (isoelectric point at pH 4–5), obtained under acidic or alkaline conditions, respectively.

The primary structure of the gelatin macromolecule is a polypeptide α-chain obtained as a result of collagen destruction [20]. Accordingly, gelatin retains all features of the amino acid composition of collagen. The gelatin macromolecule contains both positively and negatively charged groups, as well as hydroxyl moieties and hydrophobic radicals. Approximately one-third of the amino acids of the gelatin α-chain are represented by glycine Gly, which is a part of repeating tripeptide Gly–X–Y, where X most often is proline Pro and Y is hydroxyproline Hyp. The Gly–X–Y triads in the polypeptide chains of gelatin play the main role in the formation of collagen-like triple helices [21], making gelatin capable of thermo-reversible formation of physical hydrogels.

Compared with gelatin from mammals, fish gelatin is characterized by a lower content of proline and hydroxyproline [22]. This is especially true for gelatin obtained from cold-water species. This leads to deterioration in the gelling ability of fish gelatin, a decrease in gelation and melting temperatures, a decrease in gel strength [23,24,25], and increased consumption of gelatin as a food component for hydrogel formation. Many studies have been devoted to finding ways to eliminate these serious disadvantages by treating fish gelatin with various physical, enzymatic, and natural cross-linking agents [26,27,28,29,30,31]; irradiation in various frequency ranges [32,33]; high pressure [34]; enzymatic modification [35,36,37]; and additions of mono- and disaccharides [38,39]. However, the most effective and common way to improve gelling ability and rheological characteristics are the modification of fish gelatin with natural polysaccharides [40,41,42,43]. 

In the present study, we focus on the adoption of underused marine resources for different modern technologies. The main goal of this study is to characterize the structure and properties of laboratory-made fish gelatin from cod skin by comparison with known commercial gelatins of fish and mammalian origin.

## 2. Results and Discussion

### 2.1. Hydrogel Morphology by SEM

**Figure 1:**
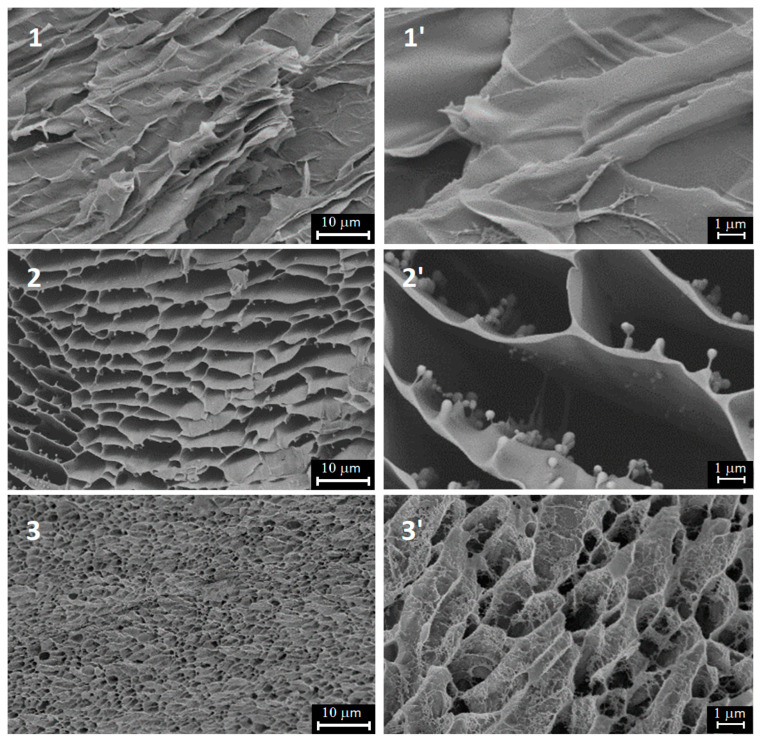
SEM images at two resolutions of gelatin xerogels. From top to bottom: Sigma pork gelatin (**sample 1**), Sigma fish gelatin (**sample 2**) and laboratory-extracted gelatin (**sample 3**).

The SEM results presented in Figure 1 depict the morphology of the studied gelatins. It is clear that the obtained structures are the result of the freeze-drying procedure of the initial gelatin solutions. However, the xerogel structure determines the initial morphology of gelatine hydrogels and can serve as the evidence of a closer gelatin network in gelatin extracted from cod skin (sample 3). As can be seen, the cellular structure of both fish gelatins differs dramatically from the mammalian one, which has extremely irregular architecture. Nevertheless, the gel structure of our laboratory-extracted cod gelatin seems to be sufficiently dense compared to that of commercial fish gelatin, being closer in this characteristic to mammalian gelatin.

### 2.2. Amorphous-Crystalline Nature of Gelatins via PXRD

**Figure 2:**
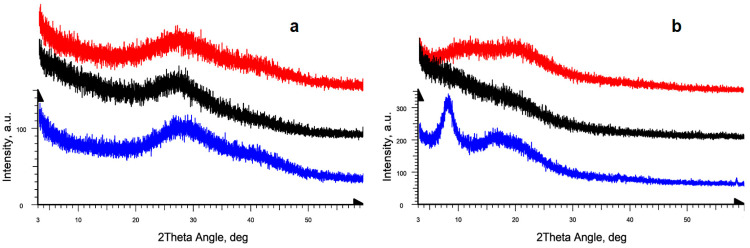
X-ray diffraction patterns of the original gelatin hydrogels (**a**) and of the dried gelatin samples (**b**): pork Sigma gelatin (blue), fish Sigma gelatin, (black), laboratory-extracted fish gelatin from cod skin (red). For clarity, the curves are shifted along the intensity axis.

The powder X-ray diffraction (PXRD) experiments showed the difference of the crystalline structures of the studied gelatines. Gelatin hydrogels were studied during their natural drying at room temperature on the surface of a silicon plate. All three studied gelatin samples in the gel state are characterized by the same type of liquid-type diffraction pattern in the form of a highly broadened amorphous halo in the range of angles 2θ ≈ 20–45°, in which two maxima can be distinguished at 28° and 40°, corresponding to the average interatomic distances in gels (Figure 2a). Note that the studied samples of fish gelatin are more “liquid” and did not show stable gel at room temperature. During hydrogel drying their diffraction patterns change noticeably that reflects changes of the protein structure upon transition from jelly-like to xerogel state, and they are also different from the gelatin samples of different origins.

The X-ray diffraction patterns of the mammalian gelatin upon drying, instead of showing the previously noted amorphous diffuse halo, show two diffraction peaks at angles 2θ = 8° and 18° (Figure 2b, black curve). These two peaks are the characteristic peaks of the triple collagen-like helix structure [44] and correspond to epy amorphous-crystalline nature of gelatin [45,46]. It was generally accepted [47] that the broad peak at 2θ ≈ 18–21° corresponds to the crystallinity of gelatins and the sharp peak at 7–8° corresponds to the residual triple helix of native collagen.

For the Sigma fish gelatin, the X-ray diffraction pattern obtained after drying of hydrogel does not show the amorphous diffuse liquid-type peak with a maximum in the angle range 2θ ≈ 27–28°. In addition, neither the 8° peak of the residual triple helix of the native collagen, nor the characteristic peak of gelatin at 2θ = 18° were clearly observed in the diffraction curve (Figure 2b, red curve); one can only notice some extended “shoulder” in the indicated angular range.

For the laboratory-extracted fish gelatin from cod skin, the initial stage of gel drying shows the same changes in the diffraction pattern. In the final stage of hydrogel drying, a change occurs in the diffraction pattern. This change is associated with the appearance of two strongly broadened diffraction peaks at angles 2θ ≈ 8–10° and 18° (Figure 2b, blue curve) with approximately equal relative intensity. The absence of the peak in the 8° region in the diffraction pattern may indicate a noticeable decrease in the content of triple helices in the fish gelatin samples compared to the mammalian gelatin and, as a consequence, different changes in its secondary structure during the formation of xerogel. This is also consistent with the visual evidence of weaker gel formation for the fish gelatin samples.

In all cases, the observed changes during drying turn out to be reversible for all three gelatin samples. When water is added to the surface of dried gels, the initial state of the samples is restored. It is reflected in the change of corresponding diffraction patterns. Moreover, such transformations can be carried out many times, but no noticeable changes in the diffraction pattern are observed if the dried sample has been kept for a long time. It indicates the absence of changes in the nature and the intensity of non-covalent interactions within the structure of the gelatin and unchanged degree of crystallinity of gelatin domains.

### 2.3. NMR Relaxation and Self-Diffusion

Nuclear magnetic resonance (NMR) is a powerful instrument to analyze molecular structure and dynamics in different systems and physical states [48,49,50,51]. The translation self-diffusion coefficient (D), spin–lattice (T_1_) and spin–spin (T_2_) relaxation times of water can indicate its interactions with protein and display protein structural states [52,53], including some peculiarities of protein supramolecular organization, for example, as a result of protein jellification [54]. The mobility of water in the polymer networks is determined by the presence of great number of macromolecules and interfaces arising from the network structure, which constrain the mobility of water molecules through intermolecular interactions, hydrophobic effect and hydrogen bonding [55,56].

**Figure 3:**
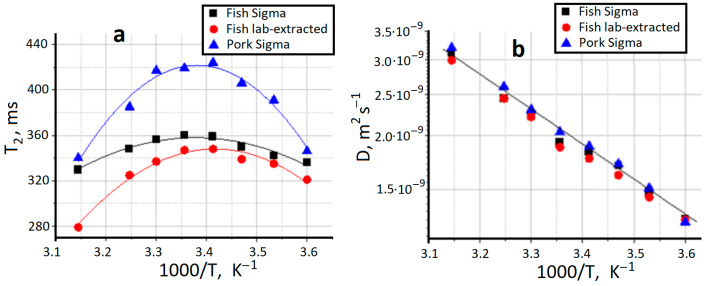
Water spin-spin relaxation time (**a**) and water self-diffusion coefficient (**b**) in gelatin hydrogels.

Figure 3 presents the results of NMR experiments on water in the three studied hydrogels. Earlier it was demonstrated [57] that valuable information about the mobility of water in hydrogels can be extracted from NMR T_2_ relaxation data using a model which takes into account the influence of chemical exchange on the T_2_ values. It was shown that the motion of water in hydrogels can be characterized by considering the presence of two distinct classes of water molecules with motions in different time scales due to the effect of binding to polymer, but which are in rapid exchange in the NMR time scale and giving the average experimental T_2_ [58]. The obtained T_2_ data (Figure 3a) indirectly demonstrate some structural differences between the studied gelatin hydrogels, resulting in different T_2_ values. However, all three gelatin systems depict the analogous temperature behavior evidently connected with the same thermal effects. Unfortunately, we could not observe the confined water behavior in the gelatin network. The study of water self-diffusion coefficient as function of diffusion time [56] did not show any decrease in D even at the maximum observation time 2000 ms. It means that the restricting boundaries for water translational mobility is not less than 140 micrometers. Apparently, the cell structure observed in CEM experiments for the xerogel state (Figure 1) is “transparent” for water transfer. It can also be seen from data presented in Figure 3b, that the water diffusive mobility is lower than that of pure water (2.6 × 10^−9^ m^2^s^−1^ at 20 °C) but its activation enthalpy 15–16 kJ/mol is close to the bulk water one.

### 2.4. Dielectric Response of Gelatin Hydrogels

**Figure 4:**
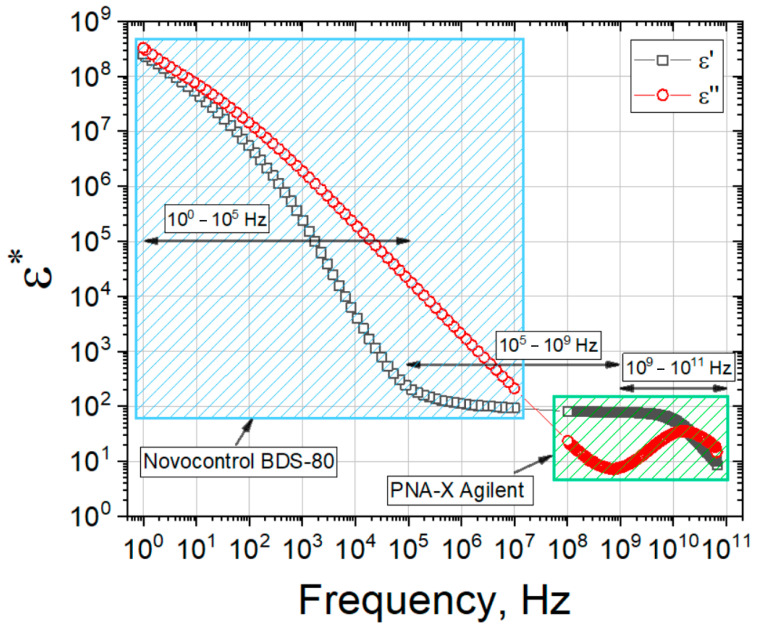
Real and imaginary parts of the dielectric spectrum of gelatin 3 measured in two frequency ranges covered summarily 10^0^–10^11^ Hz at 20 °C.

Figure 4 shows the result of the joint (real and imaginary parts) spectra of the gelatin number 3 in the frequency range 10^0^–10^11^ Hz at 20 °C. As it can be seen from Figure 4, at low frequencies 10^0^–10^4^ Hz, a process corresponding to the electrode polarization and conductivity is observed, while the gelatin relaxation processes are observed at high frequencies 10^5^–10^9^ Hz. In the frequency range of 10^9^–10^11^ Hz the relaxation of water occurs.

**Figure 5:**
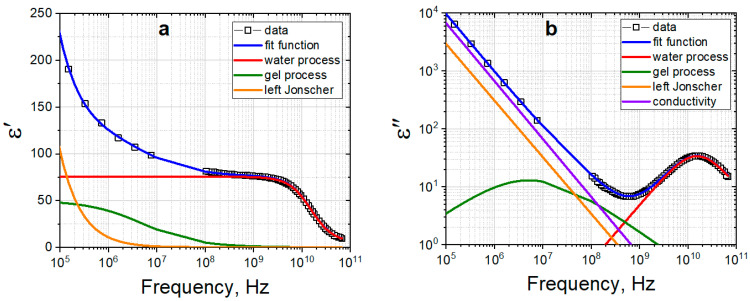
Real (**a**) and imaginary (**b**) parts of the dielectric spectrum of laboratory-made gelatin hydrogel at 20 °C. Empty square symbols show experimental data. Blue line shows fitting function, red line is process corresponding to solvent (water), green line to hydrogel component, orange line is the process of electrode polarization, purple line is conductivity.

To analyze the dielectric relaxation processes of gelatin solution, it is enough to analyze the real (Figure 5a) and imaginary (Figure 5b) parts of dielectric spectrum of gelatin hydrogel in the frequency range of 10^5^–10^11^ Hz. To take into account the electrode polarization processes [59], the Jonsher function is used. The hydrogel spectrum was approximated by Equation (1), which is a superposition of two Cole-Cole functions, conductivity contribution and Jonsher function:

(1) $$\varepsilon *(\omega )=\varepsilon_{\infty }+\frac{\Delta \varepsilon_{gel}}{1+{\left(i\omega \tau_{gel}\right)}^{\alpha_{gel}}}+\frac{\Delta \varepsilon_{water}}{1+{\left(i\omega \tau_{water}\right)}^{\alpha_{water}}}+\frac{\sigma }{i\omega \varepsilon_{0}}+A{\left(i\omega \right)}^{n-1},$$

where Δε is the increment of dielectric constant, τ is the relaxation time, α is the distribution coefficient of relaxation times (0 < α < 1), $\varepsilon_{\infty }$ is the high-frequency dielectric constant, σ is conductivity, ε_0_ is the dielectric constant of vacuum, ω is circular frequency, *A*, *n* are the amplitude and exponent of Jonsher function.

In the common case the obtained dielectric results in a wide frequency range for all three types of studied gelatins manifest themselves as the sum of four relaxation deposits. The last of these, the Jonsher function, is usually used for fine fitting of complicated relaxation data in different polymer systems. It supports to obtain other relaxation terms more accurately but has no exact physical meaning by itself [60]. Thus, we used only the first three terms of Equation (1) for further analysis. These terms are connected with the relaxation processes of gelatin molecules, relaxation of water and deposite from electric conductivity. According to the main goal of this work, to detect the similarities and difference between gelatins, we did not analyse deeply the molecular motives of relaxation behavior.

**Figure 6:**
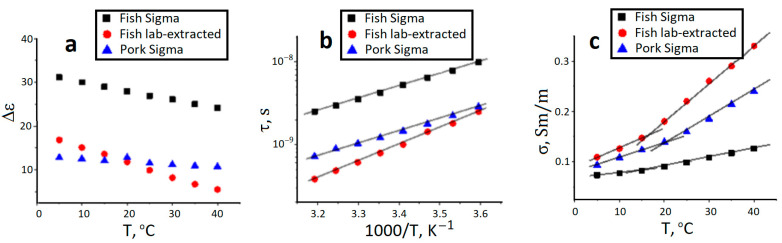
Amplitude (**a**), relaxation time (**b**) and conductivity contribution (**c**) of gelatin dielectric process.

Figure 6 presents the amplitude (Figure 6a), relaxation time (Figure 6b) and conductivity data (Figure 6c) for dielectric relaxation of gelatins studied. In general, the dielectric relaxation of the polymer systems is governed by overall rotation of polymer molecules, local segmental motions of their chains and fluctuation of bound counter-ions [61,62]. At high temperatures gelatin chains have the coil conformation [61]. Thus, the rotation of gelatin molecules and polarization of submolecular groups in the coil conformation would contribute to the dielectric relaxation of gelatin. The temperature decrease causes the lattice/helix enhancement that, in turn, may induce the chain association and spatial network formation. The formation of cross-linking junctions between helix structures would determine the relaxation behavior at low temperatures. Unfortunately, we can only see the temperature transition between these two states only on the conductivity plots. It may be the consequence of the fact, that not every phase transition can be necessarily detected as the step-wise transition of different relaxation characteristics.

However, the analysis of the dielectric phenomenon in gelatin hydrogels leads us to the conclusion that the laboratory-extracted fish gelatin from cod skin is closer to pork gelatin than to the commercial fish one from their structural point of view. It can be even seen from the detected transition on the conductivity graph—it is just sharp as for the mammalian gelatin, differing seriously from its commercial “relative”.

### 2.5. Differential Scanning Calorimetry Study of Gel Melting

**Figure 7:**
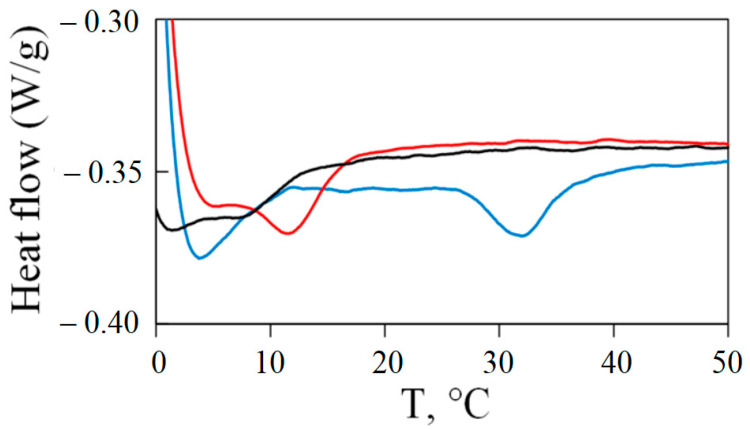
DSC curves of gels of porcine gelatin (blue line), Sigma fish gelatin (black line) and laboratory-extracted fish gelatin (red line).

The melting point, which characterizes the thermal stability and strength of the structural network under increasing temperature, is an important characteristic of gelatin hydrogels. Figure 7 shows the DSC curves of the studied gelatin gels. The endothermic peaks presented in all obtained thermograms. Their temperature positions correspond to the visual melting points of gels observed using a device for visual determination of the melting temperature (Electrothermal IA 9100 Melting Point Apparatus). For porcine gelatin (sample 1) the transition was observed in the range of 28 to 37 °C. The peak temperature was 31.9 °C and the melting enthalpy was 12 J/g (with respect to the mass of the solid gelatin sample). For extracted fish gelatin (sample 3) the transition was observed between 9 and 16 °C, peak temperature was 11.9 °C, and enthalpy of melting was about 7 J/g. The Sigma fish gelatin (sample 2) has even lower gel melting point with the peak temperature at 8.4 °C and melting enthalpy is about 3 J/g.

The obtained melting data indicates a much higher thermal stability of the porcine gelatin gel (sample 1). In addition to the higher melting temperature, it has a higher enthalpy of melting. Most likely, it indicates the higher content of proline residues and a greater number of cross-links between peptide chains in mammalian gelatins [63,64]. Interesting, comparing both fish gelatins, one can see that Sigma fish gelatin (sample 2) forms a less thermally stable gel in comparison with our laboratory sample 3.

### 2.6. Rheological Data

**Figure 8:**
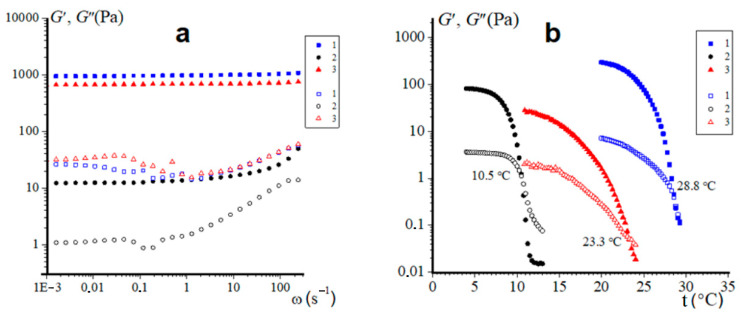
Dependencies of storage modulus G′ (full points) and loss modulus G″ (open points) (**a**) on frequency ω at γ = 1%, t = 4 °C and (**b**) on temperature t at f = 1 Hz, γ = 1% of gelatin hydrogels (6.67 wt%) from: porcine skin (sample 1), 2—cold water fish skin Sigma (sample 2) and laboratory-extracted from cod skin (sample 3).

The results of dynamic tests of gelatin hydrogels in the periodic vibration mode are presented in Figure 8. This rheological method is widely used to assess the linear and nonlinear properties of viscoelastic materials [65,66], which include physical biopolymer hydrogels [67,68] and, in particular, the gelatin gels studied [69].

Frequency dependencies of the real (storage modulus) *G*′(ω) and the imaginary (loss modulus) *G*″(ω) components of complex elastic modulus were obtained in the linear region (γ = 1%) (Figure 8a). The loss modulus *G*″ for all studied samples steadily increases in the region ω > 1 rad/s, but up to the last studied value ω (240 rad/s) does not exceed *G*′.Thus, all the studied hydrogels demonstrate the solid-like behavior, typical for physical gels in the range of stresses up to the yield stress σγ [70,71]. For the hydrogels of pork gelatin (sample 1) and extracted cod fish (sample 3) the dependences *G*′(ω) demonstrate almost constant values of the storage modulus *G*′ (quasi-equilibrium module on the plateau *G*′_pl_) throughout the studied range of ω (from 0.001 to 240 rad/s). Moreover, the *G*′_pl_ values of these samples are quite large: 940 and 670 Pa, respectively, which indicate their pronounced elastic properties. The hydrogel from fish Sigma (sample 2) has a constant *G*′_pl_ value only up to the frequency of about 1 rad/s, then *G*′ begins to steadily increase. Moreover, we have for this sample *G*′_pl_ = 12.2 Pa, which is almost two orders of magnitude lower than the similar characteristic for hydrogels of commercial mammalian gelatin and fish gelatin extracted by us from cod skin.

It should be noted that the gel-sol transition of gelatin-containing aqueous systems is a well-studied thermos-reversible physical process with well-developed research methodology [1,72]. Figure 8b shows temperature dependencies of the storage modulus *G*′(t) and loss modulus *G*″(t) of gelatin hydrogels. The temperature at the intersection points of given dependences *G*′(t) = *G*″(t) corresponds to conditions of the gel–sol transition and is equal to the melting temperature T_m_ of the hydrogel [68,69,73]. Experimental data clearly shows that hydrogels of gelatin of mammalian origin demonstrate rather high melting temperatures 28.8 °C, which corresponds to the beginning of the DSC melting peak (see Section 2.5). On the contrary, the Sigma brand fish gelatin hydrogel exhibits low thermal stability; it is characterized by a low melting point of 10.5 °C, which also falls to its melting range, determined by DSC method. The gelatin obtained from cod skin demonstrates slightly higher melting temperature (23.3 °C) than its melting range according DSC, but is still less thermally stable than the pork gelatin gel. Hence, these data (Figure 8b) correlate well with results of differential scanning calorimetry (Figure 7) and the viscoelastic properties (Figure 8a) of hydrogels.

## 3. Conclusions

The study of the World Food Program shows that the total number of undernourished people in the world is approaching to a billion. One of the ways to address this issue is to increase of the foodstuff production from alternative sources. The underused protein and polysaccharide marine resources can play crucial roles to help in nutrition, medicine, pharmacy, novel biotechnologies, etc. The main difficulty of successfully utilizing natural proteins and polysaccharides is their specific characteristics and the need of fine purification on a large scale. Biopolymers obtained from organisms living in different marine conditions can vary in chemical composition, structure and contain different impurities, which may change their physicochemical and nutritional properties for novel formulations.

In recent years, there has been a significant increase in production of gelatin from alternative sources, such as raw fish materials. The main goal of this study was to show the possibilities of experimental approaches to characterize the structure and properties of gelatins of different natural origins, namely the laboratory-made fish gelatin from the cod skin in comparison with the known commercial gelatins of fish and mammalian origin. In the present research we have combined a broad number of complementary physical-chemical methods to make the overlook of gelatin hydrogels of traditional and novel sources and technologies. In this work we have compared the morphology, supramolecular structure and colloid properties of two commercial, mammalian and fish, gelatins with gelatin extracted from cold water cod skin in the laboratory conditions. We have shown that by many parameters our experimental fish gelatin is much closer to the mammalian one. By comparing both fish gelatins it is shown that laboratory-extracted cod gelatin is essentially more thermally stable in comparison with its commercial analog. This makes it closer to the mammalian one by its rheological properties. Our previous experience in the field of complex hydrogel systems based on the interaction of gelatin with different marine polysaccharides, various metal cations and carbon nanomaterial [74,75,76,77,78,79,80] will help in the design of novel systems with broad technological potentials. 

## 4. Materials and Methods

### 4.1. Materials

**Figure 9:**
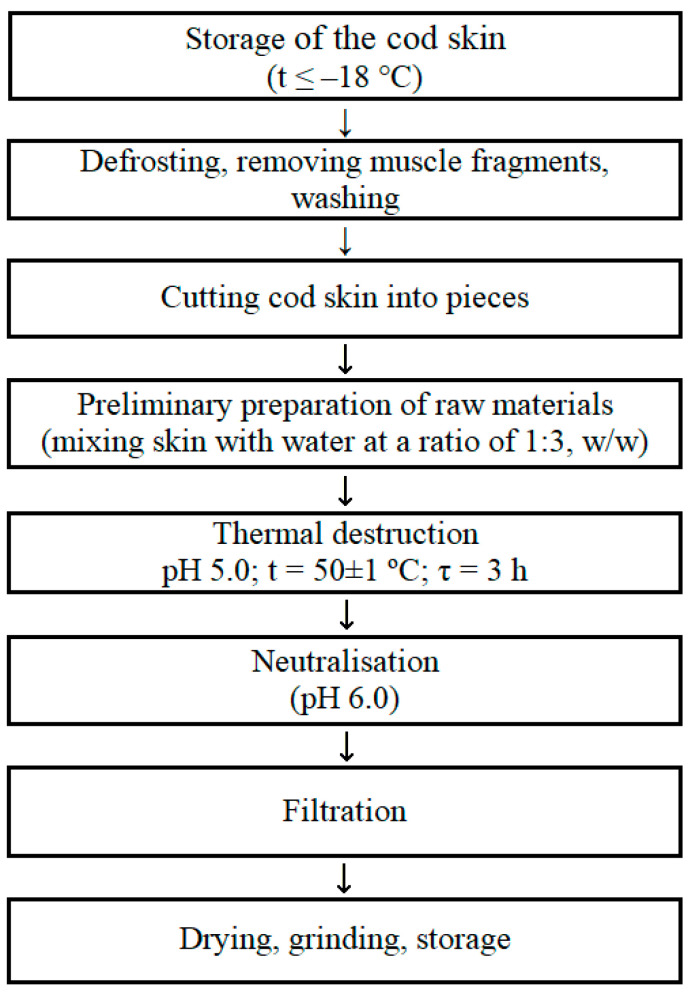
Basic technological scheme for obtaining fish gelatin from cod skin by thermal destruction method in acidic media.

In the present research we used three gelatins of type A. They are (1). gelatin from porcine skin, 90–110 Bloom (Sigma—Switzerland, G6144, Lot # BCBR5299V), (2). gelatin obtained from the skin of cold-water fish (Sigma—Canada, G 7041, Lot #S LCC7087) and (3). laboratory-extracted gelatin from cold water cod skin. Laboratorial gelatin was extracted from the skin of Atlantic cod following the standard procedure. Basic technological scheme for obtaining fish gelatin from cod skin by destruction in acidic media includes the following component stages: (1) Storage of the cod skin (t ≤ −18 °C); (2) Defrosting, removing muscle fragments, washing; (3) Cutting cod skin into pieces; (4) Preliminary preparation of raw materials (mixing skin with water at a ratio of 1:3, *w*/*w*); (5) Thermal destruction pH 5.0; t = 50 ± 1 °C; τ = 3 h; (6) Neutralization (pH 6.0); (7) Filtration; (8) Drying, grinding, storage (Figure 9). The cut cod skin was mixed with the distilled water at a ratio of 1:3 (*w*/*w*) and stirred for 10 min. Gelatin extraction was carried out at pH 5.0 for 3 h at the temperature of 50 ± 1 °C with constant stirring at a speed of 80–100 rpm. After extraction, the reaction mixture was neutralized to pH 6.0 and then filtered. The method of vacuum filtration at 30 °C together with the paper filter (Ekros, St. Petersburg, Russia) with a pore diameter of 12 µm (Akros, Russia) were used. The filtrate (gelatin solution) was dried in a FreeZone freeze dryer (Labconco, Kansas City, MO, USA) at −50 °C and a residual pressure of 3.0 Pa. The gelatin obtained was stored at 5 °C until further use.

To purify gelatin 3 from inorganic salts, the stepwise dialysis was performed. A 10% gelatin solution was placed in a dialysis tube and dialyzed sequentially against 5 mM EDTA solution, 10 mM phosphate buffer and distilled water. Dialysis was considered to be complete when the electrical conductivity of dialysate was equal to the conductivity of the dialyzed water.

To prepare gelatin samples in the majority of experiments we used 10% gelatin solutions in Milli-Q water purified with the “Arium mini” ultrapure water system (Sartorius, Gottingen, Germany). These solutions were prepared as follows. Initially required portions of gelatins were swelled in distilled water at 20 °C for 20 h, then they were stirred at 50 °C until being fully dissolved. This procedure allowed us to obtain homogeneous colloidal solutions (sols) of protein. The pH values gelatins 1–3 were 5.34, 5.26 and 5.40, correspondingly.

### 4.2. Methods

#### 4.2.1. Scanning Electron Microscopy

Scanning electron microscopy was used to study the morphology of freeze-dried samples of gelatin gels by means of the field emission scanning electron microscope “Merlin” (“Carl Zeiss”, Oberkochen, Germany). The surface morphology was studied at accelerating voltage of 15 kV. The electron microscopy experiments were carried out using gelatin cryogels [81] prepared as follows. To form hydrogel, the 10% gelatin solution was left overnight at +6 °C. Then samples were frozen in liquid nitrogen, snapped immediately and vacuum freeze-dried to obtain xerogels. The fractured sections were sputtered with gold for SEM observations.

The SEM experiments were carried out in the Interdisciplinary Center “Analytical Microscopy” (Kazan Federal University, Kazan).

#### 4.2.2. X-ray Powder Diffraction

The powder X-ray diffraction (PXRD) experiments were performed on the automatic Bruker D8 Advance diffractometer, equipped with the Vario attachment and Vantec linear PSD, using Cu K_α_1 radiation (40 kV, 40 mA), monochromated with the curved Johansson monochromator (λ = 1.5406 Å). Data were collected in the reflection mode with flat-plate samples. Samples were placed on the surface of standard silicon plate with zero diffraction, which reduces the background scattering. The samples were kept spinning (15 rpm) throughout the data collection. Patterns were recorded in the 2θ range between 3° and 70° with step size 0.008° and step time of 0.1–1.0 s. Several diffraction patterns in various experimental modes were collected for every sample. Data processing was performed using the EVA (Version 11) software package [82].

#### 4.2.3. NMR Spectroscopy

^1^H NMR experiments were performed using a Bruker Avance III WB 400 (Bruker, Preston, UK) NMR spectrometer with a working frequency of 400.27 MHz for ^1^H. In the self-diffusion experiments the Diff50 Pulsed Field-Gradient (PFG) probe was used. The diffusional decays (DD) were recorded using stimulated echo pulse sequences DiffDste. The duration of the 90° pulse was 8.12 μs, δ was in the range of 1.5 ms and the amplitude of g was varied from 0.04 up to 20 T·m^−1^. The recycle delay was 10 s. All measurements were carried out during cooling in the temperature range from 45 to 5 °C with a step of 5 °C. The waiting time for thermal equilibrium of sample volume was 10 min. ^1^H T_1_ and T_2_ NMR relaxation time measurements were performed with the inversion-recovery (180°-τ-90°-fid) and CPMG (90°-τ-180°-τ-echo) pulse sequences, respectively. The value of τ was equal to 2 ms with 128 number of points on relaxation decay. Data were processed using Bruker Topspin 3.5 software.

#### 4.2.4. Dielectric Spectroscopy

The dielectric experiments were carried out in two stages. In the first stage, dielectric measurements were carried out in the frequency and temperature ranges from 100 MHz to 60 GHz and from 50 °C to 0 °C (cooling), correspondingly. Dielectric spectra were measured on a PNA-X Agilent N5247A network analyzer in accordance with protocol of the built-in licensed software package Agilent 85070. The frequency range of measurements was 100 MHz–60 GHz. The temperature was maintained using a LOIP LT 900 thermostabilizer with a step 5 °C, temperature maintenance accuracy ±0.1 °C. A coaxial Performance Probe with a diameter of 10 mm was used as the measuring line. The sample exposure time at each temperature was 10 min. The dielectric parameters of the spectra were calculated in the Datama software(Version 2.0) package [83].

In the second stage measurements were carried out in the frequency range from 1 Hz to 10 MHz using the Novocontrol BDS-80 measuring complex. To combine two frequency ranges the measurements at the second stage were carried out in the temperature range from 0 °C to +50 °C. Thermostabilization was carried out using the Quatro system, and the accuracy of maintaining the temperature is ±0.5 °C.

#### 4.2.5. Differential Scanning Calorimetry

Experiments on the gel melting were performed using a 204 F1 Phoenix differential scanning calorimeter (Netzsch, Selb, Germany) calibrated by measuring the melting temperatures and enthalpies of standard compounds (Hg, In, Sn, Bi, Zn and CsCl). Gel samples 5–10 mg were heated in aluminum crucibles up to 40 °C (which is higher than the melting point of any sample), then incubated for 3 h at a temperature ~10 °C below the gel melting point (at 20 °C for sample 3—porcine Sigma gelatine, at −4 °C for sample 2—fish Sigma gelatin and at −1 °C for sample 3—laboratory-extracted gelatin from cod skin). The samples were then heated at a rate of 5 K/min to 50 °C. The DSC thermograms allowed us to determine the temperature and enthalpy of gel melting were recorded.

#### 4.2.6. Rheological Measurements

The rheological characteristics of gelatin samples (C_G_ = 6.67 wt.%) were measured at shearing deformations by the Physica MCR 302 rheometer (Anton Paar GmbH, North Ryde, Austria) using the cone-and-plate measuring system CP50-1. Diameter of the plate was 50 mm, the angle between the cone and plate was 1°, and the gap between the cone apex and the plate was 0.1 mm.

Measurements were carried out in the following deformation modes: periodic oscillations at constant temperature (4 °C) with varying amplitude γ or stress σ at constant frequency ω or varying frequency, the range of γ was 0.01–1000% and ω was 0.001–300 s^−1^; temperature scanning at γ = 1% and ω = 6.28 s^−1^ at increasing temperature with the rate of 1 °C/min; shearing of samples at 4 °C in the rate controlled ($\dot{\gamma }$) or stress-controlled (σ) modes in the range of 0.1–20 s^−1^ and 2–2000 Pa, respectively. The variation of given temperature was within ±0.1 °C. The relative error in measuring apparent viscosity and the components of the dynamic modulus did not exceed 10%. 

## Data Availability

Data are contained within the article.
